# What can ANS signals tell us about motor learning? An implication for better assessment of cognitive contribution to motor learning

**DOI:** 10.3389/fnbeh.2025.1715460

**Published:** 2025-10-24

**Authors:** Atsushi Yokoi

**Affiliations:** ^1^Center for Information and Neural Networks, Advanced ICT Research Institute, National Institute of Information and Communications Technology, Suita, Japan; ^2^Graduate School of Frontier Biosciences, Osaka University, Suita, Japan

**Keywords:** autonomic nervous system (ANS), cognitive, motor learning, explicit, implicit, cognitive contribution, contextual inference

## Abstract

Motor learning is supported by both explicit and implicit processes. A central question in the field of motor control is how these two processes interact and, critically, how each process can be assessed in an unbiased manner. In this perspective paper, we propose that the autonomic nervous system (ANS) offers an informative window into explicit cognitive processes during motor learning. We first briefly review studies outside the motor learning domain, where ANS activity has been linked to internal cognitive states such as surprise and uncertainty. We then discuss how these ANS-related states can be leveraged to assess the manifestation and influence of explicit processes during motor learning, as well as to explore cognitive computations that may involve central ANS activity, including contextual inference.

## Introduction

Motor learning is supported by both explicit and implicit forms of learning. While this statement seems self-evident when considering complex motor skills, such as playing a piano sonata, where explicit knowledge of musical notes, melody, and practice strategies (e.g., chunking) is important, it becomes less clear in the context of simpler motor tasks, like reaching to grasp a bottle of wine or hitting a ball with a racket. Although these actions remain complex from a biomechanical perspective, requiring coordination across multiple muscles and joints to move an end-effector from an initial to a target position in space, it is difficult to explicitly describe or implement control over each muscle, especially when accounting for complex multi-joint dynamics solving equations of motion (Hirashima et al., 2008; Kurtzer et al., 2008, 2009; Tanaka and Sejnowski, 2013). Moreover, adaptations to ongoing internal or external changes (e.g., muscle fatigue, or changes in the weight or shape of a tool) are handled almost entirely implicitly. Indeed, studies in amnesic patients have shown that learning of such tasks (e.g., pursuit rotor task, mirror drawing, reach adaptation) is preserved despite damage to the declarative memory system (Brooks and Baddeley, 1976; Shadmehr et al., 1998; Squire, 2009), reinforcing the view that motor learning is fundamentally an implicit process. As a result, much of the field, particularly in computational motor neuroscience, has focused on implicit learning processes, using relatively simple, multi-joint tasks such as reaching as model behaviors (Shadmehr et al., 2010; Wolpert et al., 2011).

However, recent studies in the same field have increasingly emphasized the role of explicit cognitive processes in motor learning (Krakauer et al., 2019; Tsay et al., 2024). This growing body of research highlights that explicit learning mechanisms significantly contribute even to relatively simple motor tasks, such as reaching movements (Bond and Taylor, 2015; Krakauer et al., 2019; McDougle et al., 2016; Tsay et al., 2024). One form of explicit strategy appears at the level of action selection or decision-making, for instance, aiming deliberately away from a target to compensate for predictable external disturbances like visuomotor rotation (Taylor et al., 2014; Taylor and Ivry, 2012). A hallmark of such explicit adjustments is that they rapidly disappear once participants are instructed to stop using the strategy or detect that the perturbation is no longer present (Benson et al., 2011; Morehead et al., 2015). This stands in stark contrast to implicit learning, in which behavioral changes, such as shifts in reach direction, can persist even when participants are fully aware that the perturbation has been removed (Morehead et al., 2015; Shadmehr et al., 2010). Here, implicit learning is more like a gradual, experience-driven modification of low-level sensorimotor mappings (or internal forward models), rather than a deliberate adjustment of the movement goal itself. Thus, our tentative definition of explicit motor learning processes in the current paper is any adaptive behavioral change that does not fall into sensorimotor recalibration (change in internal model).

Given this, key questions now concern how to objectively quantify the contributions of each learning process to observed behavior, how explicit learning is initiated, and how explicit and implicit processes interact. Currently, the assessment of explicit learning component relies in large part on verbal report by participants (Bond and Taylor, 2015; McDougle et al., 2016; Miyamoto et al., 2020). Therefore, the most important issue is how to quantify explicit contributions without interfering with the learning process itself, as frequent reports during learning can bias participants’ strategies (Maresch et al., 2021a,b). Relatedly, it is also not clear *a priori* that how much of explicit cognitive processes contributing to motor behavioral change is ready for verbalization. It is likely that the strategic re-aiming is one of many forms for explicit cognitive contribution in motor adaptation which work on top of the implicit sensorimotor recalibration. Some of them could be difficult to verbalize and other may express even without conscious awareness. Under such circumstances, there is the growing need for non-verbal methods to assess the cognitive contributions to motor learning.

In this perspective, we propose that the autonomic nervous system (ANS) may offer a promising window into explicit cognitive processes during motor learning. This is not to suggest that ANS activity serves as a direct readout of explicit learning components (e.g., aiming direction), but rather that it may reflect internal states that influence the deployment of such explicit strategies. A growing body of research in cognitive psychology and psychophysiology has used peripheral ANS indices, such as pupil diameter, skin conductance, and heart rate, to monitor participants while they perform various cognitive tasks. These studies have yielded important insights into how both peripheral ANS signals and central ANS-related brain networks contribute to cognitive processes, including error detection, performance monitoring, action selection, and decision-making (Critchley, 2005; De Berker et al., 2016; Hajcak et al., 2003; Joshi and Gold, 2020; Nassar et al., 2012; Ullsperger et al., 2014; van der Wel and van Steenbergen, 2018; Zénon, 2019).

In the following section, we first briefly review studies outside the domain of motor learning, in which ANS activity has been linked to various internal states relevant to cognitive tasks. We also highlight ANS responses to conscious/unconscious errors. Next, we present findings from motor learning research that emphasize cognitive contributions even in seemingly simple actions like reaching movements and suggest similarity between motor and cognitive learning paradigms. Finally, we discuss how ANS-related internal states can be leveraged to assess the expression and influence of explicit processes during motor learning, as well as to explore common cognitive computations across cognitive and motor tasks that may engage central ANS mechanisms.

## Autonomic nervous system activity reflects internal cognitive states

Beyond its well-known association with emotional states and threat responses (Canon, 1915; Roelofs and Dayan, 2022), ANS indices, such as pupil diameter and heart rate, have been widely used in cognitive psychology and neuroscience to probe internal state changes during non-motor (or minimally motor) cognitive tasks. For example, tasks involving mental arithmetic or working memory elicit sympathetic upregulation, reflected in increased pupil diameter (Ahern and Beatty, 1979; Beatty, 1982; Hess and Polt, 1964; Kahneman and Beatty, 1966), cardiovascular responses such as elevated heart rate and blood pressure, as well as heightened muscle sympathetic nerve activity (Anderson et al., 1987; Callister et al., 1992). These ANS responses tend to scale with the (presumably subjective) difficulty of the task, suggesting that they covary with cognitive effort (van der Wel and van Steenbergen, 2018).

Autonomic indices also respond to unexpected or unfamiliar events, reflecting their involvement in novelty detection and attentional or vigilance processes, often described as orienting responses (Sara and Bouret, 2012; Sokolov, 1990). For example, in oddball paradigms, where participants respond to or passively observe sequences of frequent and infrequent stimuli, pupil diameter and skin conductance responses (SCR) increase more strongly in response to infrequent (i.e., rare) stimuli (Bach et al., 2008; Friedman et al., 1973; Graham and Clifton, 1966; Hirano et al., 1994; Qiyuan et al., 1985), while heart rate shows deceleration. These effects often habituate with repeated stimulus presentation, highlighting the role of the ANS as an indicator of novelty detection. Particularly, the locus coeruleus (LC), one of subcortical autonomic centers, shows typical responses to novel stimuli/situations and also habituation in animals (Hervé-Minvielle and Sara, 1995; Vankov et al., 1995) and humans (Meissner et al., 2024; Murphy et al., 2014).

Related to stimulus unexpectedness, similar orienting-like ANS responses are also observed after participants made an error during various cognitive tasks, including increased pupil diameter, heightened skin conductance, and transient heart rate deceleration (Danev and de Winter, 1971; Di Gregorio et al., 2024; Hajcak et al., 2003; Maier et al., 2019; Murphy et al., 2016; Nassar et al., 2012; Preuschoff et al., 2011; Wessel et al., 2011). The post-error slowing (PES), an increase in response time after an error (Botvinick et al., 2001), and subsequent behavioral adjustments have been associated with these ANS responses, such that the magnitude of pupil dilation following an error correlates positively with PES and improved accuracy on subsequent trials (Maier et al., 2019; Murphy et al., 2016). Although this positive association between PES and post-error performance is not always observed (Wessel, 2017), error-related ANS responses likely form part of a broader performance monitoring system that coordinates post-error adjustments and behavioral control (Botvinick et al., 2001; Ullsperger et al., 2014; Wessel, 2017). An interesting topic in this context is that whether the ANS indices also respond to errors that does not reach conscious awareness. Although evidence is mixed, several studies have reported the modulation of ANS response by whether or not the participants were aware of the error (O’Connell et al., 2007; Wessel et al., 2011). Correspondingly, activity in the anterior insular cortex (AIC), one of cortical regions influencing autonomic regulation (Beissner et al., 2013), is known to be modulated by the error awareness (Klein et al., 2007; Ullsperger et al., 2010). Another recent report demonstrated that the pupil responds to novel stimulus transition pattern that participants were not aware of (Alamia et al., 2019). Although one needs to carefully interpret these results, as the definition of error (or surprise) varies across the studies, these results suggest the potential of ANS indices to probe into implicit error processing.

Building on prior work linking ANS indices to cognitive effort and orienting, more recent studies have interpreted ANS activity, particularly pupil dilation, through the lens of information theory (Zénon, 2019) and Bayesian inference frameworks that estimate hidden task or environmental states (De Berker et al., 2016; Nassar et al., 2012; O’Reilly et al., 2013; Preuschoff et al., 2011; Urai et al., 2017). For example, in decision-making and choice tasks, cue-evoked pupil dilation increases in response to both stimulus uncertainty (De Berker et al., 2016; Satterthwaite et al., 2007; Urai et al., 2017) and response uncertainty (Muller et al., 2019; Richer and Beatty, 1987), indicating that ANS activity may also reflect subjective uncertainty (Dayan and Yu, 2006; De Berker et al., 2016). Furthermore, the link between ANS indices (pupil diameter) and learning during value-based inference tasks has also been suggested. For example, Nassar et al. (2012) recorded pupil diameter while participants performed a predictive inference task in which they estimated the mean of noisy stimuli sampled from distributions that switched unpredictably. The authors developed a normative Bayesian model of belief updating that included two key latent variables: change-point probability and belief uncertainty. They found that tonic (baseline) pupil diameter correlated with belief uncertainty, while phasic outcome-evoked pupil dilation tracked the change-point probability. Notably, the learning rate (defined as the ratio of estimate update to prediction error) was jointly predicted by these two latent variables, and thus, by pupil measures. In short, large prediction errors indicate a likely change in the generative environment (i.e., distribution switch), leading to increases in both change-point probability (indexed by phasic pupil dilation) and belief uncertainty (indexed by baseline pupil diameter), which together drive an adaptive increase in learning rate. Other studies have similarly suggested that ANS activity tracks hierarchical Bayesian inference processes in volatile environments (De Berker et al., 2016; Vincent et al., 2019).

Crucially, brain regions implicated in performance and conflict monitoring, cognitive flexibility, and hierarchical Bayesian inference, such as the anterior cingulate cortex (ACC) and anterior insula cortex (AIC), also exert strong regulatory influence on ANS activity (Beissner et al., 2013; Botvinick et al., 2001; Critchley, 2005; Critchley et al., 2003; Soltani and Izquierdo, 2019; Ullsperger et al., 2014). These cortical regions, including several subcortical structures like the amygdala and LC, are called the central autonomic network (CAN) (Beissner et al., 2013). These regions have been shown to encode unsigned prediction errors and uncertainty (or volatility) of task environment in human fMRI studies (Behrens et al., 2007; Critchley et al., 2001; Loued-Khenissi et al., 2020; Preuschoff et al., 2008; Singer et al., 2009). Thus, as a result, the peripheral ANS indices, such as pupil diameter, skin conductance and heart rate, can also reflect these variables. Of these ANS indices, pupil diameter is attracting increasing attention as an established peripheral indicator of central noradrenergic activity in the LC (Joshi et al., 2016; Joshi and Gold, 2020; Rajkowski et al., 1993). The LC–noradrenaline (NA) system has been extensively studied in relation to prefrontal attention (Arnsten et al., 2012; Sara, 2009), cognitive flexibility (Aston-Jones et al., 1994; Bouret and Sara, 2004; McBurney-Lin et al., 2022), novelty detection (Bouret and Sara, 2005; Hervé-Minvielle and Sara, 1995; Vankov et al., 1995), and memory formation and consolidation (Clewett et al., 2020, 2025; Clewett et al., 2018; Sara et al., 1999; Sara and Devauges, 1988; Strange et al., 2003; Strange and Dolan, 2004; Takeuchi et al., 2016). For instance, LC neurons increase their firing rates, mirroring increases in reaction time, when animals (re-)adapt to cue–reward contingency in reversal learning tasks (Aston-Jones et al., 1997). Similarly, increases in baseline pupil diameter have been shown to precede exploratory behavior in humans performing analogous tasks (Gilzenrat et al., 2010; Jepma and Nieuwenhuis, 2011). These body of evidence, thus, suggest the involvement of CAN in various cognitive functions.

## Cognitive contribution during motor learning tasks

In the context of motor skill learning, such as sequential finger tapping, the relationship between explicit and implicit aspects of learning (e.g., declarative knowledge of the sequence versus implicit motor memory of finger transitions) has been extensively studied and debated (Abrahamse et al., 2013; Robertson, 2007; Verwey et al., 2010; Willingham et al., 1989). However, the role of explicit cognitive processes has received comparatively less attention in the domain of motor adaptation, such as goal-directed reaching movements (Shadmehr et al., 2010; Shadmehr and Krakauer, 2008; Wolpert, 2015; Wolpert et al., 2011). Although earlier work had suggested a contribution of cognitive factors in motor adaptation (Fernandez-Ruiz et al., 2011; Kagerer et al., 1997; Saijo and Gomi, 2010; Sakaguchi et al., 2001; Slachevsky et al., 2001, 2003; Taylor and Ivry, 2012; Taylor and Thoroughman, 2007, 2008), it is only recently that the field has begun to explicitly isolate and characterize the role of explicit cognitive processes in adaptation paradigms (Bond and Taylor, 2015; McDougle et al., 2015; Taylor et al., 2014).

The core idea is that behavioral changes in response to novel environments, such as force fields or visuomotor rotations, cannot be fully explained by implicit adaptation (sensorimotor recalibration) alone, but instead include a sizable contribution from explicit processes, such as deliberately altering the aiming direction to counteract perceived errors (Jakobson and Goodale, 1989; Redding and Wallace, 2006). This perspective led researchers to directly ask participants to report their intended aiming location on each trial. The reported aim was then subtracted from the observed reach direction to estimate the contribution of implicit adaptation (Bond and Taylor, 2015; McDougle et al., 2015; Taylor et al., 2014). These studies revealed a characteristic pattern: an initial, transient rise and fall in deliberate aiming responses, followed by a gradual, sustained increase in implicit adaptation across repeated trials with perturbed feedback (Bond and Taylor, 2015; McDougle et al., 2015; Taylor et al., 2014).

A very similar pattern of transient rise and fall has also been observed in reaction time (RT) following the onset of a perturbation (Benson et al., 2011; Fernandez-Ruiz et al., 2011; Haith et al., 2015; Huberdeau et al., 2015). Importantly, such transient increases in RT are positively correlated with the use of deliberate strategies in motor adaptation tasks (Fernandez-Ruiz et al., 2011; McDougle et al., 2015). Furthermore, when participants are forced to initiate movements under strict time constraints, the explicit component of adaptation is diminished (Fernandez-Ruiz et al., 2011; Haith et al., 2015), supporting the link between RT increase and strategic involvement. Also, as described in the previous section, such increase in RT following an error reminds us of the PES (Botvinick et al., 2001; Hajcak et al., 2003; Murphy et al., 2016; Ullsperger et al., 2014), which possibly implies the involvement of similar cognitive control processes triggered by sudden increase in movement error.

Computationally, the trial-by-trial trajectory of explicit strategy use closely resembles the memory dynamics predicted by two-state state-space models of learning, which posit the coexistence of fast and slow learning systems with distinct learning and forgetting rates (Smith et al., 2006). Accordingly, the fast and slow processes have been mapped onto explicit strategy use and implicit learning, respectively (McDougle et al., 2015). Intriguingly, experimental manipulations that interfere cognitive processes, such as limiting reaction time or introducing dual-task interference resulted in selective impairment of the fast component (Haith et al., 2015; Keisler and Shadmehr, 2010), strengthening the view that fast cognitive learning system and slow implicit learning system coexist in motor adaptation. These results are in line with the observation that the patients with prefrontal damage show deficit in the strategy use and correctly describe (or notice) perturbations during visuomotor adaptation (Slachevsky et al., 2001, 2003), indicating the critical contribution of performance monitoring and cognitive control systems in motor adaptation tasks.

In terms of the interaction between the explicit and implicit motor learning systems, evidence suggests something like competitive cooperation. Although the two processes work together to compensate the error produced by perturbation (McDougle et al., 2015; Miyamoto et al., 2020; Taylor et al., 2014), a number of studies have suggested the suppression of the implicit learning by the explicit process (Albert et al., 2022). For instance, a high reliance on explicit strategies may reduce the extent of implicit adaptation, as error-driven learning is attenuated when behavioral errors are actively corrected by cognitive means (Benson et al., 2011; Fernandez-Ruiz et al., 2011). Conversely, several studies have shown that implicit adaptation is enhanced when participants are unaware of the error or environmental change, for instance, when the perturbation is introduced gradually (Benson et al., 2011; Kagerer et al., 1997; Neville and Cressman, 2018; Sakaguchi et al., 2001). The exact mechanism behind such reciprocal interaction is still unclear.

Although directly asking participants to report their aiming direction on each trial initially appeared to be a simple yet effective approach for quantifying explicit learning process, recent work has raised concerns that such explicit commitment to reports may themselves influence the learning process. Indeed, several studies have demonstrated that the act of reporting can significantly bias the contribution of the explicit process (Maresch et al., 2021a; Maresch et al., 2021b), and that the standard subtraction method used to isolate implicit learning may not accurately capture the learning process (‘t Hart et al., 2024). In addition, it is not clear *a priori* that how much of cognitive processes contributing to motor behavioral change is accessible for conscious report. It is likely that the strategic re-aiming is one of many forms for explicit cognitive contribution in motor adaptation that work on top of the implicit sensorimotor recalibration. Some of them could be difficult to verbalize and other may express even without conscious awareness. Under such circumstances, there is the growing need for less biased, more ecologically valid methods to assess the cognitive contributions to motor learning (de Brouwer et al., 2018).

## How can ANS be useful for motor learning research and what does it capture?

As discussed, a common trigger for both the ANS-indexed cognitive processes and the use of cognitive strategies in motor adaptation is presumably (un)conscious^1^ detection of an error and its unexpectedness (surprise). Therefore, conceivably, the ANS responses to movement errors can provide informative markers of explicit cognitive processes during motor learning. Several recent studies have begun to test this hypothesis directly by measuring ANS indices during motor learning tasks (Nogami et al., 2025; O’Bryan and Song, 2025; Pfalz et al., 2025; Yokoi and Weiler, 2022). Consistent with the findings from cognitive inference tasks (e.g., Nassar et al., 2012), these studies suggest that phasic pupil dilation in response to movement errors reflects sensory surprise, while tonic (baseline) pupil diameter likely track subjective uncertainty about the task environment (Pfalz et al., 2025; Yokoi and Weiler, 2022). The study also suggested that the tonic pupil diameter may explain the degree to which individuals were aware of environmental change (Yokoi and Weiler, 2022). Additionally, a clear dose-dependency of phasic ANS responses (pupil dilation, skin conductance change, and heart rate deceleration) to different magnitudes of unexpected motor errors were demonstrated (Nogami et al., 2025). These observations strongly suggest that the large deviation from the expected sensory outcome leads to strong activation of ANS and implies the involvement of cognitive control process, possibly mediated by CAN, during motor learning tasks. Taken together, although still much research is needed, these findings underscore the potential of ANS measurements as non-invasive information source to study cognitive processes in human motor learning research.

What would be the “common cognitive processes” indexed by ANS activity for cognitive and motor learning tasks? In the rest of this perspective, we suggest that the contextual inference (Heald et al., 2023a; Heald et al., 2023b) would be one candidate for such cognitive computations. Contextual inference has gained increasing attention as a unifying computational principle that enables flexible behavior across domains. Rather than relying on fixed mappings between stimuli and responses, the brain appears to infer latent “contexts,” or hidden states of the environment, to appropriately guide perception and decision-making (Mante et al., 2013; Okazawa and Kiani, 2023), memory updating (Gershman et al., 2014), and motor control and learning (Heald et al., 2021). In the domain of motor learning, recent work by Heald et al. (2018, 2021) demonstrated that humans can learn and express multiple motor memories by inferring which context is currently active. This framework is powerful enough to account for a wide range of motor learning phenomena, including spontaneous recovery, interference, savings, dynamic learning rates, and the use of explicit strategies, that have been previously treated as distinct features (Heald et al., 2021). The creation and switching between multiple internal models based on the current context estimate is somewhat consistent with the reciprocal relationship between explicit strategy and implicit learning component, as well as the rapid disengagement of explicit strategy when instructed. Assuming the implicit learning system, presumably in the cerebellum, as a “base model” within this array of models (Oh and Schweighofer, 2019; Taylor and Ivry, 2014), could also explain the suppression of the implicit learning by explicit strategy (i.e., other model) through the process of context-dependent expression/suppression of specific internal model.

Similar principles apply in cognition, where behavior depends on integrating current sensory input with beliefs about hidden environmental states and their transitions (Heald et al., 2023a). Computational models such as the Hierarchical Gaussian Filter (HGF) (Mathys et al., 2011, 2014), the Volatile Kalman Filter (VKF) (Piray and Daw, 2020), and the Dirichlet Process Kalman Filter (DP-KF) (Gershman et al., 2014) formalize this process by representing beliefs not only about the current state but also about the latent state as well as its volatility, enabling dynamic modulation of learning rates and behavioral flexibility. Despite differences in implementation across these models, the problems of hierarchical inference they aim to solve are highly similar, making the contextual inference framework a potential candidate for a domain-general computational principle in the brain. Also important is that in the absence of *a priori* sensory cues that indicate specific context, the (large) prediction error is the critical cue for context change, making room for ANS to take part in this process.

Crucially, as discussed in the previous section, several key regions of the central autonomic network (CAN) (Beissner et al., 2013), including the ACC, AIC, orbitofrontal cortex (OFC), and the LC, are consistently implicated in higher-order processes such as the computation of prediction errors (i.e., surprise), uncertainty, and state transitions (Aston-Jones and Cohen, 2005; Behrens et al., 2007; Chan et al., 2021; Critchley et al., 2001; Preuschoff et al., 2008; Schuck et al., 2016; Singer et al., 2009; Soltani and Izquierdo, 2019). As repeatedly proposed (Bouret and Sara, 2005; Sara, 2009; Sara and Bouret, 2012), the LC is well-positioned to receive surprise and uncertainty signals from CAN areas and broadcast interrupt signals to multiple systems across the brain, including the hippocampus and cerebellum. The LC receives input from medial prefrontal regions such as the ACC and subcortical structures like the amygdala, and projects broadly, including to the hippocampus (Joshi and Gold, 2020; Szabadi, 2013), making it a core node for complex hidden-state learning, such as estimating environmental changes (De Berker et al., 2016; Nassar et al., 2012) or identifying episodic memory boundaries, through the hippocampus (Clewett et al., 2020, 2025). While several researchers have studied the effect of LC projection (NA) on motor learning in rodents (Heron et al., 1996; Tan et al., 1991; Watson and McElligott, 1984), a clear mechanistic view of how LC-NA system affects motor learning is still missing (Waterhouse et al., 2022).

Taken together, these bodies of evidence suggest that the CAN may support computations essential for contextual inference across cognitive and motor domains. By continuously integrating external sensory input with internal belief states, these regions enable the brain to form predictions about latent environmental structures and adjust behavior accordingly. Thus, ANS indices may offer an informative window into latent computational variables relevant to the contextual inference process in both cognitive and motor learning. In this context, revisiting prior ANS findings through the lens of the Bayesian contextual inference framework may be fruitful (Zénon, 2019).

## Conclusion and future directions

In this perspective, we have provided a brief overview of studies investigating ANS-tagged internal states during cognitive tasks and suggested their possible link to recent evidence for cognitive contributions to motor adaptation. While the proposed similarity may initially appear superficial, the concurrent rise of hierarchical inference models in both cognitive and motor neuroscience suggests that similar computational processes operate across these domains. As one of such cognitive computational processes, we further suggested that the framework of contextual inference may provide a unifying perspective to understand learning in both fields, which emphasizes conscious error detection, inference about latent task states, and selection of appropriate actions based on inferred context. The overlap between CAN and brain regions involved in hierarchical inference process further highlights the importance of ANS signals to probe these processes. Thus, we propose that the ANS can be useful for motor learning study to look closer into such process.

As already mentioned, to what degree the consciously unperceived error and corresponding ANS responses have an impact on the above inference process is still elusive. Although, clearly, the size of prediction error is an essential factor for hierarchical inference, regardless of the awareness to the error (Gershman et al., 2014; Heald et al., 2021; Mathys et al., 2011; Piray and Daw, 2020), the role of conscious awareness and ANS states in the hierarchical inference about task environment needs to be tested in the future.

Another important future question is whether the ANS is mere a leaking signal of “higher” cognitive computational processes, or it has a bidirectional influence on the cognitive processes. Converging evidence suggests that within ANS the LC-NA system is “causally” involved in task performance. Rodent studies have shown that optogenetic LC activation can shift global cortical excitability in a stimulation specific manner (i.e., tonic or phasic) (Grimm et al., 2024) and improve cognitive flexibility (Glennon et al., 2019; McBurney-Lin et al., 2022). In parallel, human studies have also demonstrated that manipulation of pupil-linked arousal by task-unrelated surprizal stimuli influenced the learning rate in an inverted-U manner (Nassar et al., 2012) and that self-regulation of pupil-linked arousal through biofeedback affected oddball detection (Meissner et al., 2024). This line of evidence supports the idea that LC-NA system (and ANS) is functionally engaged in regulating cognitive functions, such as cognitive flexibility and novelty detection, rather than a simple readout of the control process. This is also critical to the question of how we can test the hypothetical relationship between cognitive contribution in motor learning (e.g., contextual inference) and ANS in more direct way (see Box 1). Also, although we did not cover the possibility of ANS directly affecting the implicit learning in the current manuscript, direct modulation of the cerebellar complex spike rate possibly through the noradrenergic input from the LC has been reported (Carey and Regehr, 2009; Sun et al., 2019). This topic is open for future research.

BOX 1 **Experimental paradigms to probe autonomic contributions to context formation in motor learning.**

The autonomic nervous system (ANS) may serve as a key interface linking cognitive and motor learning processes. We propose that *the formation of context boundaries and the creation of new motor memories depend on ANS-tagged novelty or surprise signals*. To test this “ANS–context–motor” hypothesis, coordinated efforts across human and animal studies are essential.

**Human paradigms**

Although invasive neural manipulation in humans is limited, behavioral flexibility enables systematic examination of how autonomic arousal relates to context transitions during motor learning. Forward- and back-translation of paradigms, such as reach adaptation involving contextual inference, and reversal learning, allow quantification of how trial-by-trial changes in ANS activity accompany the emergence of new internal models. Causal manipulations of autonomic state are accessible through physiological means (e.g., cold pressor test (Antov et al., 2015), isometric handgrip), pharmacological modulation of adrenergic or cholinergic tone (Jepma et al., 2016, 2018), transcutaneous vagus nerve stimulation (tVNS) (Kilgard et al., 2025), or voluntary biofeedback (e.g., pupil-size training) (Meissner et al., 2024). When combined with simultaneous ANS monitoring and non-invasive neural recordings (EEG, MEG, fMRI), these approaches can test whether autonomic activation precedes or gates neural signatures of context re-encoding.

**Animal paradigms**

In animal models, circuit-specific manipulation techniques (optogenetics, chemogenetics) allow causal probing of ANS–context interactions at cellular resolution. Motor learning tasks such as reach adaptation in rodents (Mathis et al., 2017) or marmosets (Ebina et al., 2018, 2024) provide quantitative readouts of boundary formation. AI-based kinematic tracking, such as the DeepLabCut (Nath et al., 2019) enables simultaneous assessment of movement adaptation and peripheral ANS signals, including contactless indices such as nasal temperature (Kuraoka and Nakamura, 2022; Nakayama et al., 2005). Direct autonomic manipulations, including pharmacological, implanted vagus nerve stimulation (Collins et al., 2021; Mridha et al., 2021), or locus coeruleus photostimulation (Glennon et al., 2019; Grimm et al., 2024; McBurney-Lin et al., 2022), can determine whether perturbing ANS-linked arousal circuits alters the delineation of motor contexts.

**Cross-species integration**

Aligning task structures and ANS metrics across species will reveal whether autonomic signals merely co-vary with, or actively define, the cognitive boundaries that segment motor memories.

In summary, on top of its bodily functions, ANS signals could allow us to better understand flexible, adaptive human behavior in an uncertain world.

## Data Availability

The original contributions presented in this study are included in this article, further inquiries can be directed to the corresponding author.

## Footnotes

1 As discussed in the previous section, several studies have reported ANS responses to errors show modulation by whether the participants were aware of the error. For LC and pupil, clear responses to noxious stimuli (e.g., foot shock) have been reported in anesthetized animals (Ohsawa et al., 1997; Sugiyama et al., 2012), suggesting that conscious detection may not always be required for some ANS responses. Therefore, there might be a continuum of this process between conscious and unconscious error detection. Nevertheless, it is certain that a large error elicits both of ANS responses and conscious awareness.
